# A Systematic Review of the Effects of Bisphosphonates on Osteoblasts In Vitro

**DOI:** 10.1007/s00223-025-01390-w

**Published:** 2025-06-16

**Authors:** Henrique Hadad, Laís Kawamata de Jesus, Maísa Pereira da Silva, Maria Eduarda de Freitas Santana Oliveira, Fernando Pozzi Semeghini Guastaldi, Ola Nilsson, Roberta Okamoto, Francisley Ávila Souza

**Affiliations:** 1 Division of Oral & Maxillofacial Surgery, Department of Diagnosis and Surgery, School of Dentistry, São Paulo State University (UNESP), Araçatuba, São Paulo Brazil; 2 Division of Pediatric Endocrinology and Center for Molecular Medicine, Department of Women’s and Children’s Health, Karolinska Institutet and Karolinska University Hospital, Visionsgatan 18, L8:01, Solna, 171 76 Stockholm, Sweden; 3 Department of Oral and Maxillofacial Surgery, Massachusetts General Hospital, Harvard School of Dental Medicine, Boston, MA USA; 4 Department of Basic Sciences, School of Dentistry, São Paulo State University (UNESP), Araçatuba, SP Brazil

**Keywords:** Bisphosphonates, Osteoblast, Osteoclast, Bone biology

## Abstract

Bisphosphonates (BPs) are widely used to treat bone disorders, prevent skeletal-related events, and manage bone metastasis. These drugs are synthetic analogs of pyrophosphate and primarily function by inhibiting osteoclast activity. However, increasing evidence suggests that they also have an effect on osteoblasts. This systematic review aims to evaluate how bisphosphonates affect osteoblasts by summarizing findings from in vitro studies on the impact of BPs on osteoblast lineage cells, addressing the following question: “*Do bisphosphonates affect osteoblast cell lineage function?*”. For this purpose, the PICO framework was followed, and 36 articles were selected for inclusion in this review. The data suggest that the molecular mechanisms in osteoblasts can vary depending on the specific type of bisphosphonate, as well as the concentration and duration of treatment, leading to either stimulation or inhibition of osteogenesis. Additionally, studies have shown that certain BPs, such as zoledronic acid, can interfere with osteoblast differentiation, proliferation, gene expression, and mineralization capacity, potentially impairing bone healing. On the other hand, other drugs, such as alendronate, demonstrate more positive effects on cell function. Some drugs, such as pamidronate and clodronate, exhibited mixed effects; however, it was observed that high concentrations of these drugs can lead to cytotoxic effects. Despite these adverse effects, it is important to recognize that the clinical benefits of managing bone disorders often outweigh the potential risks highlighted in this review.

## Introduction

The body’s bones constitute a multifunctional organ, serving roles in mineral homeostasis, protection, and locomotion [1, 2]. Bone integrity relies on continuous remodeling, which involves the resorption of old bone and the formation of new bone tissue through the coordinated action of osteoclasts and osteoblasts, thereby maintaining bone homeostasis [3, 4]. One of the main axes of bone remodeling control is the RANK-RANKL-OPG pathway (receptor activator of NF-κB, receptor activator of NF-κB ligand, and osteoprotegerin). This pathway controls osteoclastogenesis, regulates calcium metabolism, and mediates the interaction between osteoblast and osteoclast activity [5–7].

Focusing on the treatment of conditions characterized by excessive osteoclast-mediated bone resorption, a class of antiresorptive drugs has emerged as a primary option for managing these conditions. Known as bisphosphonates (BPs), these drugs are approved for treating various skeletal disorders, including osteoporosis, Paget’s disease of bone, hypercalcemia, and for the prevention of bone metastasis and other skeletal-related events (SREs) in multiple myeloma and solid malignancies. By inhibiting osteoclast activity [8, 9], BPs offer considerable clinical benefits [10, 11].

However, the use of BPs has been associated with adverse effects, such as medication-related osteonecrosis of the jaw (MRONJ) and nephrotoxicity [12–14], raising questions about their mechanisms of action, broader effects, and the development of these drugs beyond their impact on osteoclasts [15]. Divided into non-nitrogen-containing bisphosphonates (NN-BPs), such as etidronate and clodronate, and nitrogen-containing bisphosphonates (N-BPs) classes, such as zoledronate, alendronate, and others, based on their different modes of molecular structure, they exhibit differential potency and mechanisms of action [16].

According to Coxon et al. (2008) [17], osteoclasts are the only bone cells capable of releasing and internalizing BPs. Beyond their interaction with calcium, BPs also affect hydroxyapatite (HA) by slowing the transition from amorphous to crystalline HA [18–22]. Nevertheless, there is growing concern about their impact on osteoblasts [23–25]. Recent data suggest that the use of BPs, particularly zoledronic acid (ZA), can impact the migration and viability of endothelial cells, fibroblasts, and osteoblasts [26–29]. Furthermore, data have demonstrated that BPs can induce cytotoxic effects on osteoblasts, inhibiting osteogenesis, reducing mineralization capacity, and exhibiting anti-angiogenic properties. [30, 31] Additionally, studies both in vitro [25, 32, 33] and in vivo [34, 35] have demonstrated that these drugs may exhibit a dose-dependent effect.

In summary, there seems to be a controversy regarding the effects of BPs on osteoblast-like cells, which may be influenced by the specific BP, its concentration, the cell model, and the experimental conditions. This review aims to synthesize the effects of BPs on osteoblasts, with a focus on the underlying molecular and cellular mechanisms. It seeks to highlight how BPs impact the function and survival of osteoblasts, as well as explore their implications in bone homeostasis and tissue remodeling.

## Methods

### Protocol and Registration

**Fig. 1:**
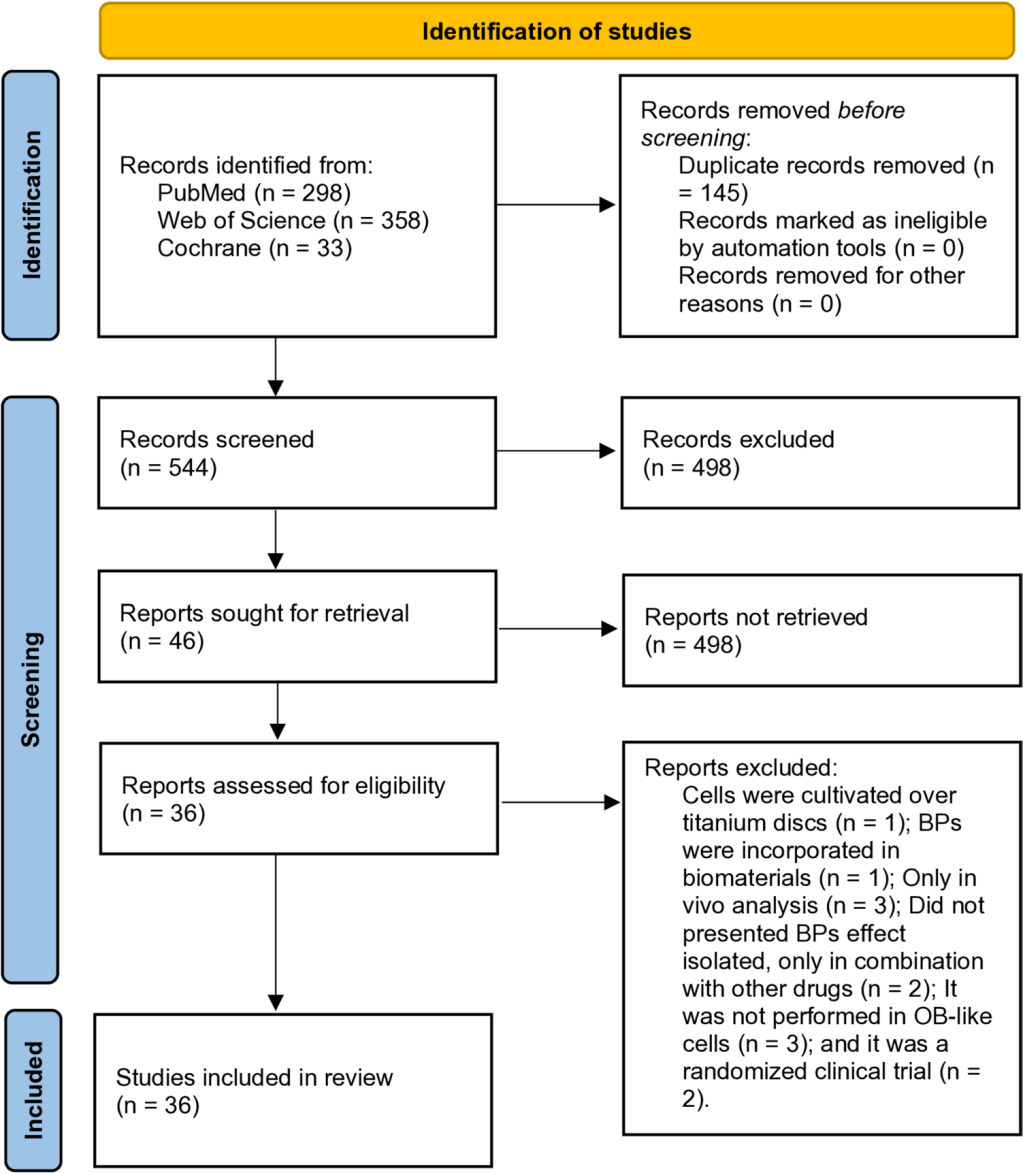
PRISMA flow diagram illustrating the search strategy and selection process for the studies included in this review

This systematic review was registered in the Open Science Framework (osf.io/z529e). It was based on the Preferred Reporting Items for Systematic Reviews and Meta-Analyses (PRISMA) (Fig. 1) [36] checklist structure and followed the recommendations of the Enhancing the Quality and Transparency of Health Research Network (EQUATOR Network).

### Focused Question

This systematic review was conducted to answer the following question: “*Do bisphosphonates affect osteoblast cell lineage function?*”.

### Search Strategy

To achieve this, a literature search was conducted across three databases (PubMed, Web of Science, and Cochrane Library), without time restrictions, but limited to publications in the English language. The search utilized MeSH terms and entry terms as follows: “(osteoblast OR osteoblast-like cell line) AND (bisphosphonates OR zoledronic acid OR alendronate OR risedronate OR etidronate OR ibandronate OR clodronate OR tiludronate OR pamidronate OR zoledronate) AND (control OR non-treatment) AND (viability OR toxicity OR gene expression OR growth OR migration OR apoptosis OR proliferation).” An independent manual search was also performed using terms tailored for each database, including gray literature and relevant journals in the field. The manual search also included reviewing the reference lists of pertinent review studies. Alerts were set up for each database to maintain an up-to-date search strategy.

### Eligibility Criteria

We followed the PICO framework [4033] for this review to address our focused question.

(P) population: osteoblast or osteoblast-like cell lineage.

(I) intervention: administration of bisphosphonates (such as zoledronic acid, alendronate, risedronate, etidronate, ibandronate, clodronate, tiludronate, pamidronate, or zoledronate).

(C) comparison: untreated cells or control group.

(O) outcome: in vitro analysis related to cell function, such as viability, proliferation, growth, migration, apoptosis, toxicity, and markers for gene expression.

For this purpose, the following inclusion criteria were considered: In vitro studies assessing the effects of bisphosphonates on osteoblasts or osteoblast-like cell lines. Reviews, clinical studies, technical notes, abstracts, in vivo studies, and studies not published in English were not eligible.

### Study Selection

All data from the three databases were imported into the Rayyan – Intelligent Systematic Review Platform. The screening process began by excluding all duplicate files. Subsequently, two reviewers (H.H. and L.K.J.) independently assessed all titles and abstracts based on the inclusion and exclusion criteria. In cases of disagreement, a third reviewer (FÁS) was consulted, and the final decision was made by consensus. The inter-rater agreement was calculated using the kappa coefficient. Finally, a full screening process was conducted for the papers that met the inclusion and exclusion criteria.

### Data Extraction

The following data were extracted from each paper: author(s), year of publication, type of drug, concentration, dose, period and frequency of administration, duration of the experiments, and cell linage, as well as the transcription of the outcomes observed by the authors (such as viability, proliferation, growth, migration, apoptosis, toxicity, and marker expression). One attempt was made to contact the corresponding author for missing data.

### Quality and Risk of Bias

This research used a modified version of the Systematic Review Center for Laboratory Animal Experimentation (SYRCLE) tool to address and assess the risk of bias. Many tools are currently being used to assess the quality and risk of bias; however, there is no official tool for use in in vitro studies [37, 38]. Several tools have been used in this context, some of which were proposed by the authors or adapted from validated tools such as SYRCLE.

## Results

A total of 689 articles were identified (298 from PubMed/MEDLINE, 358 from Web of Science, and 33 from Cochrane) that correlated the effects of BPs on osteoblasts. After removing duplicates, 544 publications were screened by title and abstract. A total of 47 full texts were selected. Then, after applying the inclusion and exclusion criteria, 36 articles were included in this review (Table 1).

**Table 1:** Studies characterization

Study	Cell	Drug and Posology	Time points
Giuliani et al., 1998 [64]	MG-63	ET 10^−12^ up to 10^−4^ M over 4 h	4 h
		CL 10^−10^ up to 10^−4^ M over 4 h	
		AL 10^−12^ up to 10^−6^ M over 4 h	
Mackie et al., 2001 [66]	UMR 106–01	PAM 10^−8^ up to 10^−4^ M over 4 days	1, 2, 3, 4 and 5 days
		CL 10^−6^ up to 10^−2^ M over 4 days	
Mathov et al. 2001 [65]	ROS 17/2.8	OLP 10^−8^ up to 10^−5^ M over 4 h	18 h
		PAM 10^−8^ up to 10^−5^ M over 4 h	
		ET 10^−8^ up to 10^−5^ M over 4 h	
Im et al., 2004 [60]	HOB	AL 10^−9^ up to 1^0–6^ M over 3 days	24, 48 and 72 h
	MG-63	AL 10^−12^ up to 10^−4^ M over 3 days	
		RS 10^−12^ up to 10^−4^ M over 3 days	
Pan et al., 2004 [49]	HOB	ZA 5 X 10^−6^ M over 5 day	3 and 5 days
Kellinsalmi et al., 2005 [39]	HOB	CL 10^−12^ up to 10^−3^ M over 60 s	60 s
		PAM 10^−12^ up to 10^−3^ M over 60 s	
		ZA 10^−12^ up to 10^−3^ M over 60 s	
Greiner et al., 2006 [50]	HOB	ZA 10^−5^ M over 6 days	1, 2, 3, 4, 5, and 6 days
		ZA 3 × 10^-5^ M over 6 day	
		ZA 5 × 10^-5^ M over 6 day	
		ZA 10^−4^ M over 6 day	
		ZA 1.5 X 10^−4 ^M over 6 day	
Duque et al., 2007 [57]	BMSCs (human)	AL 10^−7^ M over 14 days	7 and 14 days
		AL 10^−8^ M over 14 days	
		AL 10^−9^ M over 14 days	
Kim et al., 2009 [58]	BMSCs (mouse)	AL 3.69 × 10^ − 6^ up to 10^−4^ M over 2 days	24 or 48 h
Pozzi et al., 2009 [46]	BMSCs (human), HOB	ZA 10^− 7^ M over 21 days	7, 14, and 21 days
		ZA 5 × 10 ^− 7^ M over 21 days	
		ZA 5 × 10 ^− 6^ M over 21 days	
		ZA 10 ^− 5^ M over 21 days	
Xiong et al., 2009 [61]	MG-63	AL 10^−10^ up to 10^−6^ M over 4 days	48, 72, or 96 h
Labrinidis et al., 2009 [41]	HOB	ZA 10^−6^ up to 10^−4^ M over 72 h	72 h
Orriss et al., 2009 [40]	Calvaria-derived osteoblasts (rat)	CL 10^−9^ up to 10^−5^ M over 14 days	2, 4, 6, 8, 10, 12, and 14 days
		PAM 10^−9^ up to 10^−5^ M over 14 days	
		ZA 10^−9^ up to 10^−5^ M over 14 days	
Corrado et al., 2010 [26]	HOB	ZA 10^−10^ up to 10^−3^ M over 48 h	48 h
Koch et al., 2010 [54]	HOB	IB 5 × 10^−5^ M over 14 day	1, 2, 5, 10, and 14 days
		CL 5 × 10^−5^ M over 14 day	
		ZA 5 × 10^−5^ M over 14 day	
Koch et al., 2011 [55]	HOB	IB 10^−7^ up to 10^−5^ M over 14 day	1, 2, 10, and 14 days
		CL 10^−7^ up to 10^−5^ M over 14 day	
		ZA 10^−7^ up to 10^−5^ M over 14 day	
Koch et al., 2011 [42]	HOB	IB 5 × 10^−5^ M over 14 day	1, 2, 5, 10, and 14 days
		CL 5 × 10^−5^ M over 14 day	
		ZA 5 × 10^−5^ M over 14 day	
Walter et al., 2011 [27]	HOB	CL 5 × 10^−6^ M over 72 h	72 h
		CL 5 × 10^−5^ M over 72 h	
		CL 10^−4^ M over 72 h	
		CL 2 × 10^−4^ M over 72 h	
		CL 5 × 10^−4 ^M over 72 h	
		IB 5 × 10^−6^ M over 72 h	
		IB 5 × 10^−5^ M over 72 h	
		IB 10^−4^ M over 72 h	
		IB 2 × 10^−4 ^M over 72 h	
		IB 5 × 10^−4^ M over 72 h	
		PAM 5 × 10^−6^ M over 72 h	
		PAM 5 × 10^−5^ M over 72 h	
		PAM 10^−4^ M over 72 h	
		PAM 2 × 10^−4^ M over 72 h	
		PAM 5 × 10^−4^ M over 72 h	
		ZA 5 × 10^−6^ M over 72 h	
		ZA 5 × 10^−5^ M over 72 h	
		ZA 10^−4^ M over 72 h	
		ZA 2 × 10^−4 ^M over 72 h	
		ZA 5 × 10^−4^ M over 72 h	
Patntirapong et al., 2012 [51]	MC3T3, MSC	ZA 10^−8^ up to 10^−4^ M over 12 h	1, 3, and 7 days
Basso et al., 2013 [47]	MG-63	ZA 10^−6^ M over 21 days	7, 14, and 28 days
		ZA 5 × 10^−6^ M over 21 days	
Ishtiaq et al., 2015 [56]	MG-63, MC3T3	AL 10^−12^ up to 10^−6^ M overnight	3 days
		ZA 10^−12^ up to 10^−6^ M overnight	
Manzano-Moreno et al., 2015 [63]	MG-63	PAM 10^−4^ M over 1 day	1 day
		PAM 5 × 10^−5^ M over 1 day	
		PAM 10^−5^ M over 1 day	
		AL 10^−4^ M over 1 day	
		AL 5 × 10^−5^ M over 1 day	
		AL 10^−5^ M over 1 day	
		IB 10^−4^ M over 1 day	
		IB 5 × 10^−5^ M over 1 day	
		IB 10^−5^ M over 1 day	
		CL 10^−4^ M over 1 day	
		CL 5 × 10^−5^ M over 1 day	
		CL 10^−5^ M over 1 day	
Zara al., 2015 [43]	HOB	ZA 10^−5^ M over 5 days	5 days
		ZA 5 × 10^−5^ M over 5 days	
		ZA 10^−4^ M over 5 days	
Huang et al., 2016 [33]	MC3T3	ZA 10^−8^ up to 10^−4^ M over 7 days	1, 3, 5, and 7 days
Manzano-Moreno et al., 2016 [84]	MG-63	CL 10^−9^ up to 10^−5^ M over 2 days	1 and 2 days
Zafar et al., 2016 [44]	HOB	ZA 10^−6 ^M over 3 days	1, 2, and 3 days
		ZA 10^−5 ^M over 3 days	
		ZA 2 × 10^−5^ M over 3 days	
		ZA 3 × 10^-5^ M over 3 days	
		ZA 4 × 10^-5^ M over 3 days	
		ZA 5 × 10^-5^ M over 3 days	
Chen et al., 2017 [69]	HOB	99Tc-MDP 10^−12^ up to 10^−6^ M over 72 h	1, 2, 3, 6, and 9 days
Ma et al., 2018 [62]	BMSCs (rat)	AL 10^−6^ M over 2 days	1, 2, and 3 days
Manzano-Moreno et al., 2018 [52]	MG-63, HOB	CL 10^−9^ up to 10^−5^ M over 1 day	1 day
		AL 10^−9^ up to 10^−5^ M over 1 day	
		ZA 10^−9^ up to 10^−5^ M over 1 day	
Raudhah et al., 2018 [67]	HOB	PAM 6.49 × 10^−5^ M over 72 h	1, 3, and 7 days
Yazici et al., 2018 [48]	SAOS-2	ZA 5 × 10^−6^ M over 24 h	24 h
Huang et al., 2019 [33]	MC3T3	ZA 10^−6^ up to 10^−4^ M over 48 h	24, 48, and 72 h
Kim et al., 2019 [68]	BMM (mice), MLO-Y4	CL 10^−7^ and 10^−6^ M over 3 days	3 days
		ZA 10^−7^ and 10^−6^ M over 3 days	
Xu et al., 2019 [59]	BMM (rat)	AL 7 X 10^−3^ M over 24 h	24 h
di Vito et al., 2020 [32]	PDLSCs	ZA 10^−7^ M over 14 days	1, 5, 7, 10, and 14 days
		ZA 10^−6^ M over 14 days	
		ZA 1.5 × 10^−6^ M over 14 days	
		ZA 2 × 10^−6^ M over 14 days	
		ZA 3 × 10^−6^ M over 14 days	
		ZA 5 × 10^−6^ M over 14 days	
Hadad et al., 2023 [25]	BMSCs (human)	ZA 10^−7^ M over 3 days	7 and 14 days
		ZA 10^−6^ M over 3 days	
		ZA 5 × 10 M over 3 days	

*MG-63* osteosarcoma cells line, *UMR 106–01* osteosarcoma cells line, *HOB* human osteoblast, *BMSCs* bone marrow stem cells, *ROS 17/2.8* osteosarcoma cells line, *MSC* mesenchymal stem cells, *MC3T3* osteoblast precursor cell line derived from mouse’s calvaria, *SAOS-2* osteosarcoma cells line, *BMM* bone marrow mononuclear cells, *MLO-Y4* murine osteocyte-like cell line, *PDLSCs* periodontal ligament stem cells, *ET* etidronate; *CL* clodronate, *AL* alendronate, *PAM* pamidronate, *ZA* zoledronic acid, *IB* ibandronate, *RS* risedronate, *OLP* olpadronate, *99Tc‑MDP* Tc-99 m methylene diphosphonate, *M* molar

### Main Findings

This review encompasses 36 studies (Table 2) which explore both NN-BP’s (also known as a first-generation, such as Etidronate [ET—doses ranging from 10^−4^ up to 10^−12^ M], and Clodronate [CL—10^−2^ up to 10^–12^ M]) and N-BP’s (such as Pamidromate [PAM – 10^–3^ up to 10^–12^ M], Olpadronate [OLN—10^–5^ up to 10^–8^ M], Alendronate [AL—10^–2^ up to 10^–12^ M], Ibandronate [IB – 10^–4^ up to 10^–7^ M], Risedronate [RS—10^–4^ up to 10^–8^ M], and Zoledronic acid [ZA—10^–3^ up to 10^–12^ M]). Only one study explored a novel compound (99Tc-MDP—10^–4^ up to 10^–12^ M).

**Table 2:** Groups, outcomes, and main findings

Study	Groups	Analysis								Main Findings
		Viability	Proliferation	Mineralization (ALP activity/ Staining)	Adhesion/ Spreading		Markers (qPCR) and other analysis			
Giuliani et al., 1998 [64]	ET 10^–4^ M up to 10–12 M	–	–	–	–		ET (10^−4^ M* up to 10^−9^ M*), CL (10^−4^ M* up to 10^−7^ M*), and AL (10^–6^ M* to 10–^12^ M*) exposition inhibited IL-6^**−**^ production induced by IL-1 + TNF			BPs can act directly on human osteoblastic cells and inhibit the production of IL-6, suggesting that the inhibitory effect of these drugs on bone resorption may be mediated, at least in part, by the regulation of the production of this cytokine
	CL 10^–4^ M up to 10–12 M									
	AL 10^–6^ M up to 10–12 M									
Mackie et al., 2001 [66]	PAM 10^–8^ M	NA	–	–	–		At 10^–5^ M PAM the expression of OPG and COL1A1 remained unchanged from 0 to 5 days, RANKL and OPN* were downregulated by prolonged exposure. These effects were similar for CL (showed no result). ELISA demonstrated an increase in DNA fragmentation, cells treated with PAM (10^–9^ M up to 10^–4^ M) and CL (10^–5^ and 10^–3^) demonstrated increases* in apoptotic cells exposed to higher concentrations (PAM at 10^–4^ M and CL at 10^–3^ M)			BPs do not only act on the growth and apoptosis of cells, but also, by altering the expression of osteoclast-regulating factors, they may inhibit the activity of osteoclasts and their recruitment
	PAM 10^–7^ M	Reduced*	–	–	–					
	PAM 10^–6^ M	Reduced*	–	–						
	PAM 10^–5^ M	Reduced*	–	NA	–					
	PAM 10^–4^ M	–	–	–	–					
	CL 10^–2^ M	Reduced*	–	–	–					
	CL 10^–3^ M	–	–	–	–					
	CL 10^–4^ M	Reduced	–	–						
	CL 10^–5^ M	–	–	NA	–					
	CL 10^–6^ M	Reduced	–	–	–					
Mathov et al., 2001 [65]	OLP 10^–8^ M	–	Increased*	–	–		Western blot demonstrated increasing in ERK1/2 phosphorylation after 30 s			Cells of the osteoblastic lineage are targets of BP action
	OLP 10^–7^ M	–	Increased*	–	–					
	OLP 10^–6^ M	–	Increased	–	–					
	OLP 10^–5^ M	–	Increased	–	–					
	PAM 10^–8^ M	–	Increased*	–	–		Western blot demonstrated increasing in ERK1/2 phosphorylation after 30 s			
	PAM 10^–7^ M	–	Increased*	–	–					
	PAM 10^–6^ M	–	Increased	–	–					
	PAM 10^–5^ M	–	Increased	–	–					
	ETI 10^–8^ M	–	Increased*	–	–		Western blot demonstrated increasing in ERK1/2 phosphorylation after 90 s			
	ETI 10^–7^ M	–	Increased*	–	–					
	ETI 10^–6^ M	–	Increased*	–	–					
	ETI 10^–5^ M	–	Increased*	–	–					
Im et al., 2004 [60]	AL 10^–9^ M HOB	–	Increased	–	–		At 10^–8^ M concentration, after 72 h both AL and RS moderately increased the expression of BMP–2, COL1A1 and OCN. And competitive RT-PCR showed that AL and RD increased the cDNA copy number for OCN. And vitamin D also increased the copy number of OCN			RS and AL promoted the proliferation and maturation of osteoblasts
	AL 10^–8^ M HOB	–	Increased*	Increased	–					
	AL 10^–7^ M HOB	–	Increased	–	–					
	AL 10^–6^ M HOB	–	NA	–	–					
	AL 10^–12^ M MG-63	–	Increased*	–	–					
	AL 10^–11^ M MG-63	–	Increased*	–	–					
	AL 10^–10^ M MG-63	–	Increased*	–	–					
	AL 10^–9^ M MG-63	Increased	Increased*	Increased	–					
	AL 10^–8^ M MG-63	Increased*	Increased*	Increased*	–					
	AL 10^–7^ M MG-63	Increased	Increased*	Increased	–					
	AL 10^–6^ M MG-63	Increased*	Increased*	Increased	–					
	AL 10^–5^ M MG-63	–	Increased*	–	–					
	AL 10^–4^ M MG-63	–	Reduced	–	–					
	RS 10^–12^ M MG-63	–	Increased*	–	–					
	RS 10^–11^ M MG-63	–	Increased*	–	–					
	RS 10^–10^ M MG-63	–	Increased*	–	–					
	RS 10^–9^ M MG-63	Increased*	Increased*	Increased*	–					
	RS 10^–8^ M MG-63	Increased*	Increased*	Increased*	–					
	RS 10^–7^ M MG-63	Increased*	Increased*	Increased	–					
	RS 10^–6^ M MG-63	Increased*	Increased*	Increased	–					
	RS10^−5^ M MG-63		Increased*							
	RS 10^–4^ M MG-63		Reduced							
Pan et al., 2004 [49]	ZA 5 × 10^−6^ M	–	–	–	–		ZA decreased both transmembrane* and intracellular RANKL expression for 3 days. ZA increased OPG protein expression after 72 h* e 120 h*, increased gene expression of TACE after 2 h* and TAPI-2 partially reversed the shedding of transmembrane RANKL mediated by ZA			ZA, in addition to its direct effects on mature OCs, may inhibit the recruitment and differentiation of OCs by cleavage of transmembrane RANKL in OB-like cells by upregulating the TACE
Kellinsalmi et al., 2005 [39]	CL 10^–12^ M	–	Increased	Increased	–		Apart from 10^–12^, which ALP was increased*, the other doses do not appear to affect ALP expression			The effect of BPs to promote the proliferation and maturation of osteoblasts was not as evident
	CL 10^–9^ M	–	NA	Increased	–					
	CL 10^–6^ M	–	NA	Reduced	–					
	CL 10^–3^ M	–	Reduced	Increased*	–					
	PAM 10^–12^ M	–	Increased	Reduced	–					
	PAM 10^–9^ M	–	Increased	NA	–					
	PAM 10^–6^ M	–	Increased	NA	–					
	PAM 10^–3^ M	–	Reduced*	Increased*	–					
	ZA 10^–12^ M	–	Reduced*	Reduced	–					
	ZA 10^–9^ M	–	Reduced	Increased	–					
	ZA 10^–6^ M	–	Increased*	Increased	–					
	ZA 10^–3^ M	–	Reduced*	NA	–					
Greiner et al., 2006 [50]	ZA 10^–5^ M	NA	–	–	–		pro-COL1A1 synthesis was decreased* (3 × 10^–5^, 5 × 10^–5^, and 1.5 × 10^–4^ M). OPG and RANKL expressions were highly decreased even with 10^–5^ M			High doses of BPs reduce cell viability and modulates osteoblast function by decreasing their abilities in the mineralization phase
	ZA 3 × 10^–5^ M	NA	–	–	–					
	ZA 5 × 10^–5^ M	Reduced*	–	–	–					
	ZA 10^–4^ M	Reduced*	–	–	–					
	ZA 1.5 × 10^–4^ M	Reduced*	–	–	–					
Duque et al., 2007 [57]	AL 10^–9^ M	–	–	Increased* ALP activity	–		AL (10^−8^ M) increased* OCN expression at week 2 and cbfa1 expression was increased* at week 1. Dose–dependent effect on cellular mineralization at 10^–7^ M* and 10^–8^ M* compared with a lower concentration of 10^–9^ M after 2 weeks			Data showed a potential anabolic effect of AL in vitro through the stimulation of osteogenic differentiation of MSCs
	AL 10^–8^ M	–	–		–					
	AL 10^–7^ M	–	–		–					
Kim et al., 2009 [58]	AL 3.69 × 10^−4^ M	–	Increased	Increased	–		Flow cytometry showed CD44 expression was increased on cells treated with AL, as OCN and OPN. Microscopic analysis showed nodules of mineralization, and Alizarin Red S staining was more intense in treated with AL cells. SEM–EDX provided evidence of mineralization by the appearance of a calcium peak, and the degree of mineralization was increased by AL			These data suggest AL enhances osteogenic differentiation when treated with mouse mesenchymal stem cells in osteogenic differentiation medium
	AL 3.69 × 10^−3^ M									
	AL 3.69 × 10^−2^ M									
Pozzi et al., 2009 [46]	ZA 10^−7^ M	Reduced	–	ALP activity was reduced in a dose-depended manner	–		Alizarin red staining showed reduced osteoblast function with increasing doses of ZA after 21 days weeks. Osteoblasts differentiation showed a dose- and time-dependent toxicity			Data showed that prolonged exposure of differentiated osteoblast in vitro to ZA resulted in a cytotoxic effect compared with undifferentiated BMSC
	ZA 5 × 10^−7^ M	Reduced	–		–					
	ZA 5 × 10^−6^ M	Reduced	–		–					
	ZA 10^–5^ M	Reduced	–		–					
Xiong et al., 2009 [61]	AL 10^–6^ M	–	Increased*	Increased*	–		BMP2, OCN, and COL1A1 expression were upregulated after 96 h, being the highest with AL at 10^–8^ M. And AL stimulated calcium deposition at 10^–6^ M*, 10^–8^ M* and 10^–10^ M* on days 7, 14, and 21			AL, apart from inhibiting osteoclastic bone resorption, is also a promoter of osteoblast proliferation and maturation
	AL 10^–8^ M	–	Increased*	Increased*	–					
	AL 10^–10^ M	–	Increased*	Increased	–					
Labrinidis et al., 2009 [41]	ZA 10^–6^ up to ZA 10–4 M	Reduced *	–	–	–		Increased* expression of caspase-3 and decreasing in zVAD. ZA also led to unprenylated Rap1A			Data demonstrated that ZA can impair on cell viability and adhesion
Orriss et al., 2009 [40]	CL 10^–9^ M	–	NA	NA	–		COL1A1 expression was NA			Data demonstrated that BPs, especially ZA in higher concentration can affect osteoblast proliferation and function
	CL 10^–8^ M	–			–					
	CL 10^–7^ M	–			–					
	CL 10^–6^ M	–		Reduce MNF	–					
	CL 10^–5^ M	–			–					
	PAM 10^–9^ M	–	NA	NA	–		COL1A1 expression was NA			
	PAM 10^–8^ M	–			–					
	PAM 10^–7^ M	–			–					
	PAM 10^–6^ M	–	Reduced	Reduced MNF	–					
	PAM 10^–5^ M	–			–					
	ZA 10^−9 ^M	–	NA	NA	–		COL1A1 expression was NA			
	ZA 10^−8^ M	–	Reduced	Reduced MNF and ALP activity	–		COL1A1 expression was reduced			
	ZA 10^−7^ M	–			–					
	ZA 10^−6^ M	–			–		COL1A1 expression was inhibited			
	ZA 10^−5^ M	–			–					
Corrado et al., 2010 [26]	ZA 10^–10^ M	–	NA	NA	–		ZA increased OCN between 10^–10^ M* and 10^–5^ M* and decreased OCN synthesis between 10^–4^ M* and 10^–3^ M*. Increased cell apoptosis was observed at 10^–4^ M* and 10^–3^ M*			ZA exert different cellular biochemical effects depending on dosage and support the hypothesis that their positive effect on bone mineral density could be partially due to an anabolic action on bone forming cells
	ZA 10^–9^ M	–	NA	NA	–					
	ZA 10^–8^ M	–	NA	NA	–					
	ZA 10^–7^ M	–	Increased*	Increased*	–					
	ZA 10^–6^ M	–	Reduced*	Increased*	–					
	ZA 10^–5^ M	–	Reduced*	Increased*	–					
	ZA 10^–4^ M	–	Reduced*	Reduced*	–					
	ZA 10^–3^ M	–	Reduced*	Reduced*	–					
Koch et al., 2010 [54]	IB 5 × 10^–5^ M	–	–	–	–		Cyclin D1 expression decreased until the 6th day; however, after this, its expression increased. COL1A1 expression seems to be highly stimulated over the first 10 days, except on 14th, which was highly decreased			BPs, especially ZA, can affect proliferation of osteoblast. In general, they seemed to enhanced collagen expression, apart from ZA
	CL 5 × 10^–5^ M	–	–	–	–		Cyclin D1 expression was increased in all time points, but 2nd day. COL1A1 expression was NA over the first 6 days; however, it had increased on 10 and 14 days			
	ZA 5 × 10^–5^ M	–	–	–	–		Cyclin D1 expression was NA until 6th day; however, after this timepoint, cyclin D1 expression was decreased. COL1A1 expression was NA at 1st day; however, it was increased over time, except on 14th, which was highly decreased			
Koch et al., 2011 [55]	IB 5 × 10^–7^ M	–	–	–	–		MSX1, MSX2, DLX5, OCN, and RUNX2 expressions were NA			Data suggest that CL barely effects osteoblast differentiation, while findings suggest that at higher concentration ZA and IB can modulate osteoblast metabolism
	IB 5 × 10^–6^ M	–	–	–	–					
	IB 5 × 10^–5^ M	–	–	–	–		MSX1, MSX2, and DLX5 expressions were increased over time. However, RUNX2 expression was NA. Late enhanced upregulation (14th days) was observed for OCN expression			
	CL 5 × 10^–6^ M	–	–	–	–		MSX1, MSX2, and RUNX2 expressions were NA. Only 10^–3^ M enhanced DLX5 expression and OCN expression was NA			
	CL 5 × 10^–5^ M	–	–	–	–					
	CL 5 × 10^−3^ M	–	–	–	–					
	ZA 5 × 10^–6^ M	–	–	–	–		MSX1, MSX2, DLX5, OCN, and RUNX2 expressions were NA			
	ZA 5 × 10^–7^ M	–	–	–	–					
	ZA 5 × 10^–5^ M	–	–	–	–		MSX1, MSX2, DLX5, and RUNX2 expressions were increased over time. Late enhanced upregulation (14th days) was observed for OCN expression			
Koch et al., 2011 [42]	CL 5 × 10^–5^ M	–	–	–	Increased aVb3 integrin and NA tenascin C		–			BPs seems to affect adhesion and migration
	IB 5 × 10^–5^ M	–	–	–	Increased aVb3 integrin and tenascin C					
	ZA 5 × 10^–5^ M	–	–	–			ZA modified cell morphology (from typical dendritic to spherical appearance)			
Walter et al., 2011 [27]	CL 5 × 10^–6^ M	Reduced	–	–	–		–			Nitrogen-containing bisphosphonates (ZOL, PAM, IB) affect the viability, apoptosis and migration of the cels more than non-nitrogen-containing bisphosphonates (CL)
	CL 5 × 10^–5^ M	Reduced					Apoptosis TUNEL-Assay weak impact and Boyden migration assay (Maintained)			
	CL 10^–4^ M	Reduced*					–			
	CL 2 × 10^–4^ M	Reduced					–			
	CL 5 × 10^–4^ M	Reduced					–			
	IBAN 5 × 10^–6^ M	Reduced					–			
	IBAN 5 × 10^–5^ M	Reduced					Apoptosis TUNEL-Assay weak impact and Boyden migration assay (inhibited)			
	IBAN 10^–4^ M	Reduced					–			
	IBAN 2 × 10^–4^ M	Reduced*					–			
	IBAN 5 × 10^–4^ M	Reduced					–			
	PAM 5 × 10^–6^ M	Reduced					–			
	PAM 5 × 10^–5^ M	Reduced*					Apoptosis TUNEL-Assay greatest impact and Boyden migration assay (inhibited)			
	PAM 10^–4^ M	Reduced					–			
	PAM 2 × 10^–4^ M	Reduced					–			
	PAM 5 × 10^–4^ M	Reduced					–			
	ZA 5 × 10^–6^ M	Reduced*					–			
	ZA 5 × 10^–5^ M	Reduced					Apoptosis TUNEL-Assay greatest impact and Boyden migration assay (inhibited)			
	ZA 10^–4^ M	Reduced					–			
	ZA 2 × 10^–4^ M	Reduced					–			
	ZA 5 × 10^–4^ M	Reduced					–			
Patntirapong et al., 2012 [51]	ZA 10^–8^ M—MSC	Increased	–	Reduced *	–		Alizarin red reduced in all cells and concentrations*; RUNX2 and COL1A1 were downregulated, BSP had little effect in MSC			Inhibitory effect of ZA on cell viability, cell proliferation, and osteoblast differentiation of both MSC and osteoprogenitor cells
	ZA 10^–7^ M—MSC	Reduced	–	Reduced *	–					
	ZA 10^–6^ M—MSC	Reduced	–	Reduced	–					
	ZA 5 × 10^–6^ M—MSC	Reduced *	–	Reduced *	–					
	ZA 10^–5^ M—MSC	Reduced *	–	Reduced *	–					
	ZA 5 × 10^–5^ M—MSC	Reduced *	–	Reduced *	–					
	ZA 10^–4^ M—MSC	Reduced *	–	Reduced *	–					
	ZA 10^–8^ M—MC3T3	Reduced	–	Increased	–		Alizarin red reduced in all cells and concentrations*; RUNX2, COL1A1, and BSP had little effect in MC3T3			
	ZA 10^–7^ M—MC3T3	Reduced	–	Increased	–					
	ZA 10^–6^ M—MC3T3	Reduced	–	Increased	–					
	ZA 5 × 10^–6^ M—MC3T3	Reduced	–	Reduced *	–					
	ZA 10^–5^ M—MC3T3	Reduced	–	Reduced *	–					
	ZA 5 × 10^–5^ M—MC3T3	Reduced	–	Reduced *	–					
	ZA 10^–4^ M—MC3T3	Reduced	–	Reduced *	–					
Basso et al., 2013 [47]	ZA 10^–6^ M	Reduced*	–	ALP activity was decreased* at 14th and 21st days. Decrease in MNF specially at 7 days*	-		TPP* was decreased. At 21st day, both OCN and ALP expressions were decreased*			Cytotoxic effects were noted when ZA was used in MG-63 cells in a concentration-dependent manner. Also, mineralization capacity was decreased
	ZA 5 × 10^–6^ M		–	ALP activity was increased at 7 days but decreased* at 14th and 21st days. Decrease* in MNF overtime	–					
Ishtiaq et al., 2015 [56]	AL 10^–6^ M	–	–	–	–		In MG-63, the concentration of VEGF decreased* in AL 10^–7^ and 10^–6^; and in HCC1 concentration of VEGF decreased* in AL 10^–8^ and 10^–6^ and increased in AL 10^–10^ and 10^–7^. P1NP do not change			Nitrogen-containing BPs suppress osteoblastic production of VEGF and ANG-1, at higher, non-toxic concentrations
	AL 10^–7^ M	–	–	–	–					
	AL 10^–8^ M	–	–	–	–					
	AL 10^–9^ M	–	–	–	–					
	AL 10^–10^ M	–	–	–	–					
	AL 10^–11^ M	–	–	–	–					
	AL 10^–12^ M	–	–	–	–					
	ZA 10^–6^ M	–	–	–	–		In MG-63, the concentration of VEGF decreased* in ZA 10–9,10^–8^,10^–7^,10^–6^. The concentrations of the ANG-1 decreased in ZA 10^–11^, 10^−10^, 10^−9^, 10^−8^, and 10^–6^. P1NP did not change. VEGF mRNA decreased in ZA10^−10^,10^–8^,10^–7^ and 10^–6^. In HCC1, concentration of VEGF decreased* in ZA 10^–9^ and 10^–6^			
	ZA 10^–7^ M	–	–	–	–					
	ZA 10^–8^ M	–	–	–	–					
	ZA 10^–9^ M	–	–	–	–					
	ZA 10^–10^ M	–	–	–	–					
	ZA 10^–11^ M	–	–	–	–					
	ZA 10^–12^ M	–	–	–	–					
Manzano-Moreno et al., 2015 [63]	PAM 10^–5^ M	–	NA	–	–		While 10^–5^ M was NA, 5 × 10^–5^ M and 10^–4^ M increased* % of cells in G0/G1 phase and decreased cells in G2/M. % of apoptotic cells were increased*			High doses of BPs reduce proliferation capacity of osteoblast by arrest cell cycling and inducing apoptosis
	PAM 5 × 10^–5^ M	–	Reduced*	–	–					
	PAM 10^–4^ M	–	Reduced*	–	–					
	AL 10^–4^ M	–	Reduced*	–	–		While 10^–5^ M was NA, 5 × 10^–5^ M and 10^–4^ M increased* % of cells in G0/G1 phase, but G2/M was NA. % of apoptotic cells were increased*			
	AL 5 × 10^–5^ M	–	Reduced*	–	–					
	AL 10^–5^ M	–	NA	–	–					
	IB 10^–4^ M	–	Reduced*	–	–		While 10^–5^ M was NA, 5 × 10^–5^ M and 10^–4^ M increased* % of cells in G0/G1 phase and decreased cells in G2/M. % of apoptotic cells were increased*			
	IB 5 × 10^–5^ M	–	Reduced*	–	–					
	IB 10^–5^ M	–	NA	–	–					
	CL 10^–4^ M	–	Reduced*	–	–		While 10^–5^ M was NA, 5 × 10^–5^ M and 10^–4^ M increased* % of cells in G0/G1 phase, but G2/M was NA. % of apoptotic cells were increased*			
	CL 5 × 10^–5^ M	–	Reduced*	–	–					
	CL 10^–5^ M	–	NA	–	–					
Zara et al., 2015 [43]	ZA 10^–5^ M	Reduced	–	NA	NA		OCN* expression was lower, whereas E11/gp83 was NA. Annexin-V/PI was NA. Bax pro-apoptotic protein expression was reduced. ELISA assay reported increased* levels for COL1A1, reduced* IL-6, but NA PGE2 levels			ZA can delay osteobalstic cells differentiation
	ZA 5 × 10^–5^ M	Reduced*	–	–	–		–		–	
	ZA 10^–4^ M	Reduced*	–	–	–		–		–	
Huang et al., 2016 [33]	ZA 10^–8^ M	Increased	–	Increased	–		Col1 decreased* in 10–8 up to 10^–6^ M; ALP decreased in 10^−7^ up to 10^–6^ M; OCN decreased* in 10^–6^ M; and Runx2 decreased* in 10^–8^ up to 10^–6^ M; P-p38/p38* and P-ERK1/2/P-ERK decreased in 10^–8^ up to 10^–6^ M			ZA at higher concentrations induced cytotoxicity toward osteoblasts, and ZA at lower concentrations suppressed osteoblast differentiation by downregulating the expression of BMP2
	ZA 10^–7^ M	Increased	–	Increased	–					
	ZA 10^–6^ M	Increased	–	Increased	–					
	ZA 10^–5^ M	Reduced *	–	–	–		In the ZA 10^−7^, 10^−8^ increased the apoptosis *			
	ZA 10^–4^ M	Reduced *	–	–	–					
Manzano-Moreno et al., 2016 [84]	CL 10^–9^ M	–	Increased*	Reduced*	–		Flow cytometry shown CD54*, CD80*, CD86*, and HLA-DR* expressions were reduced			Although CL increase MG-63 cells proliferation, data suggest that also decrease their differentiation capacity
	CL 10^–7^ M									
	CL 10^–5^ M									
Zafar et al., 2016 [44]	ZA 10^–6^ M	NA	–	NA	NA		–			ZA can affect viability, proliferation and migration in a concentration-related manner. Also, apoptosis rate increase in higher concentration of ZA. PCR array demonstrated that ZA could alter both angiogenic and osteogenic gene expression
	ZA 10^–5^ M	Reduced*		MNF was not observed in any group	Increased* in caspase-3/7 and a 50% decreased * in migration in a concentration-dependent manner					
	ZA 2 × 10^–5^ M	Reduced*								
	ZA 3 × 10^–5^ M									
	ZA 4 × 10^–5^ M									
	ZA 5 × 10^–5^ M						BMP6, IGF2, PDGFβ, and EREG were upregulated, while FGFR2, COL11A1, CCL2, CXCL12, ANGPT1, and THBS1 were downregulated			
Chen et al., 2017 [69]	99Tc-MDP 10^–12^ M	–	Increased*	–	–		–			99Tc-MDP induced osteoblast proliferation and differentiation, enhanced osteoblast growth and matrix mineralization, and thus bone formation, and enhanced the osteogenic function of osteoblasts
	99Tc-MDP 10^–11^ M	–	Increased*	Increased*	–		–			
	99Tc-MDP 10^–10^ M	–	Increased*	Increased*	–		–			
	99Tc-MDP 10^–9^ M	–	Increased*	Increased*	–		–			
	99Tc-MDP 10^–8^ M	–	Increased*	Increased*	Apoptotic cells increased		Increased OPG*, OPG/RANKL* and BMP* decreased RANKL*. Fraction of cells in the G0/G1 phases reduced*			
	99Tc-MDP 10^–7^ M	–	Increased*	Increased*	–		–			
	99Tc-MDP 10^–6^ M	–	Increased*	Increased*	–		–			
	99Tc-MDP 10^–5^ M	–	Increased*	–	–		–			
	99Tc-MDP 10^–4^ M	–	Increased*	–	–		–			
Ma et al., 2018 [62]	AL 10^–6^ M	Increased *	–	Increased *	–		OCN*, Osterix*, RUNX2*, ODF*, OPG*, COL1A1*, and ALP* expressions were upregulated, and TRAP* was downregulated. Differentiation* in OB was increased. Upregulation of the IFN-β/STAT1 signaling pathway			AL improve osteogenic differentiation of BMCs toward osteoblast linage
Manzano-Moreno et al., 2018 [52]	CL 10^–5^ M	–	–	In the HOB cell line, TGF-β1 expression was decreased in 10^–5^ M. However, in the MG-63 cell line, TGF-β1 was decreased in 10^–5^ M, whereas 10^–7^ and 10^–9^ M increased TGF-β1* expression						Data suggest that regardless of the drug and concentration used, in both cell lines (HOB or MG-63), the gene expression related to growth, differentiation, and interaction between osteoclast and osteoblast were affect
	CL 10^–7^ M	–	–	RANKL* expression was decreased in 10^–5^ M for both HOB and MG-63 cell lines. However, OPG* was decreased in HOB 10^–5^ M (NA in 10^–7^ and 10^–9^ M) and increased in MG-63 (10^–5^, 10^–7^, 10^–9^)						
	CL 10^–9^ M	–	–	RUNX2*, ALP*, COL1A1*, OSX*, OSC*, BMP2, BMP7, and VEGF were reduced in the same pattern for both cell type						
	AL 10^–5^ M	–	–	In the HOB cell line, TGF-β1* expression was decreased in 10^–5^ M. However, in the MG-63 cell line, TGF-β1 was increased in AL 10^–5^ M, whereas 10^–7^ and 10^–9^ M increased TGF-β1* expression						
	AL 10^–7^ M	–	–	RANKL* expression was decreased in 10^–5^ M for both HOB and MG-63 cell lines. However, OPG* was decreased in HOB 10^–5^ M (NA in 10^–7^ and 10^–9^ M), and increased in MG-63 (10^–5^, 10^–7^, 10^–9^)						
	AL 10^–9^ M	–	–	RUNX2*, ALP*, COL1A1*, OSX*, OSC*, BMP2, BMP7, and VEGF were reduced in the same pattern for both cell type						
	ZA 10^–5^ M	–	–	For both HOB and MG-63 cell lines, 10^−5^ M was NA. However, in MG-63 cells, both 10^–7^ and 10^–9^ M increased TGF-β1* expression						
	ZA 10^–7^ M	–	–	RANKL* expression was decreased in 10^–5^ M for both HOB and MG-63 cell lines. However, OPG* was decreased in HOB 10^–5^ M (NA in 10^–7^ and 10^–9^ M) and increased in MG-63 (10^–5^, 10^–7^, 10^–9^)						
	ZA 10^–9^ M	–	–	RUNX2*, ALP*, COL1A1*, OSX*, OSC*, BMP2, BMP7, and VEGF were reduced in the same pattern for both cell types						
Raudhah et al., 2018 [67]	PAM 6.49 × 10^–5^ M	Reduced*	Increased*	–		–		Western blot demonstrated that protein level for RUNX2 and OSX increased		Rate of proliferation and expression of Runx2 and Osx in hFOB 1.19 cells treated with pamidronate has modest effect on hFOB1.19
Yazici et al., 2018 [48]	ZA 5 × 10^–6^ M	Reduced*	–	–		–		ZA increased intracellular ROS, mitochondrial depolarization, and apoptosis* (caspase-3 and -9)		ZA demonstrated a cytotoxic effect for osteoblast-like cells. This effect was augmented when ZA was associated with dexamethasone and anti-angiogenic agents
Huang et al., 2019 [33]	ZA 10^–6^ M	–	Reduced	–		–		Increased OCN expression		P21 presented as a key regulator in the transition from a proliferating osteoprogenitor to a post-proliferative osteoblast after ZOL treatment
	ZA 10^–5^ M	–	Reduced	–		–		Increased OCN expression*		
	ZA 10^–4^ M	–	Reduced*	–		–		Increased OCN expression*, decreased CDK6, OCN, β-actin (Western blot, cytometry, PCR),p21*, and p27		
Kim et al., 2019 [68]	ZA 10^–7^ M	Maintained	Increased	Reduced		–		Both doses enhanced RANKL*, Sclerostin*, M-CSF, ANG, TRAP + MCNs*, IL-6 *, Gp 130 *, p-STAT3/STAT3* expression		Zoledronate enhanced osteocyte-mediated osteoclastogenesis through elevated expression of IL-6 and subsequent RANKL expression. JAK2/STAT3 pathways seemed to be involved in zoledronate-induced RANKL expression in MLO-Y4 cells
	ZA 10^–6^ M	Maintained	Increased	Reduced*		–				
	CL 10^–7^ M	–	–	–		–		Both doses enhanced RANKL, Sclerostin, M-CSF, ANG, TRAP + MCNs, IL-6, Gp 130, and p-STAT3/STAT3* expression ,		
	CL 10^–6^ M	–	–	–		–				
Xu et., 2019 [59]	AL 7 X 10^–3^ M	Reduced OC*	Reduced OC*	Increased*		–		OPG, OCN, β-actin, PCR, expression and phosphorylation levels of PKA, STAT3, and STAT1 were increased*		Alendronate may promote osteoblast differentiation through the PKA-STAT3 and activator of transcription 1 pathway and increase osteoblast viability and activity of osteoblasts
		Increased OB*	Increased OB*							
di Vito et al., 2020 [32]	ZA 10^–7^ M	NA	–	–		–		ZA decreased* RUNX2 and COL1 expressions but enhanced OCN in a dose-dependent manner. Doses higher than 3 × 10^–6^ M increased* cell arrest (by increasing G0/G1 and decreasing S phase)		ZA doses > 1.5 × 10–6 M impaired cells viability, induced apoptosis, and can impair in osteogenic differentiation
	ZA 10^–6^ M	NA				–				
	ZA 1.5 × 10^–6^ M	Reduced*				–				
	ZA 2 × 10^–6^ M	Reduced*				Reduced*				
	ZA 3 × 10^–6^ M	Reduced*				Reduced*				
	ZA 5 × 10^–6^ M	Reduced*				Reduced*				
Hadad et al., 2023 [25]	ZA 10^–7^ M	NA	NA	Reduced		NA		RUNX2*, COL1A1, and ALP expressions were downregulated		Dose-dependent effect of ZA on the osteogenic differentiation of hBMSCs toward the osteoblast lineage
	ZA 10^–6^ M	NA	Compromised	Reduced		NA				
	ZA 5 × 10^–6^ M	Reduced*	Compromised*	Reduced*		Reduced*				

*ET* etidronate, *CL* clodronate, *AL* alendronate, *PAM* pamidronate, *ZA*, zoledronic acid, *IB* ibandronate, *RS* risedronate, *OLP* olpadronate, *99Tc‑MDP* Tc-99 m methylene diphosphonate; *M* molar, *MG-63* osteosarcoma cells line, *HOB* human osteoblast, *MSC* mesenchymal stem cells, *MC3T3* osteoblast precursor cell line derived from mouse’s calvaria, *NA* not affected, *OC* osteoclast, *OB *osteoblast, *ALP* alkaline phosphatase, MNF mineralization nodules formation, *aVb3* alpha-v beta-3 integrin, *TGF-β1* transforming growth factor beta 1, *RANKL* receptor activator of nuclear factor kappa-B ligand, *OPG* osteoprotegerin, *RUNX2* runt-related transcription factor 2, *COL1A1* collagen type 1, *OSX* osteoblast-specific transcription factor – osterix, *OSC* oxidosqualene cyclase, *MSX* msh homeobox like protein, *DLX5* distal-less homeobox 5, *BMP* bone morphogenetic protein, *VEGF* vascular endothelial growth factor; *ROS* reactive oxygen species, *OCN* osteocalcin, *mRNA* messenger ribonucleic acid, *M-CSF* macrophage colony-stimulating factor, *ANG* angiopoietin, *TRAP* tartrate-resistant acid phosphatase, *IL-6* interleukin 6, *Gp 130* glycoprotein 130, *STAT* signal transducer and activator of transcription, *PKA* protein kinase A, *BPs* bisphosphonates, *TACE* TNF-α converting TNF-alpha converting enzyme, *BMSC* bone marrow stem cells

*significantly

In general, the primary cell culture was made, in the majority, using osteoblastic cell line (mainly MG-63, but also cells line from C57BL/6 mouse’s calvaria [MC3T3-E1], human and rats bone marrow stem cells [BMSCs], rats or mice bone marrow mononuclear cells [BMMC], periodontal ligament stem cells [PDLSCs], murine osteocyte-like cell line [MLO-Y4], epithelial-like cell from Sprague–Dawley rat [UMR 106-01], osteosarcoma cell line [ROS 17/2.8, UMR-106-1, and SAOS-2], and hepatocellular carcinoma cell line [HCC1]). However, some authors also used human osteoblast cells (HOBs) or calvaria-derived osteoblasts from rats.

Overall, the effects of ZA on OB-like cells were more negative when compared to the other BPs. Studies have shown that ZA led to a reduction in viability, proliferation, adhesion, migration, and mineralization of these cells [27, 32, 39–45]. Many studies have observed a dose-dependent pattern of ZA’s effect on cells [26, 46], which increases cytotoxicity, affecting their function [33, 47], and enhances cytotoxic effects, thereby increasing intracellular ROS production and mitochondrial depolarization [48]. Osteogenesis markers, such as RUNX2, COL1A1, ALP, OCN, and β-actin, were reduced when cells were exposed to ZA in several studies [25, 49–53]. Additionally, ZA can affect the expression of proteins related to cell reproduction, decreasing the expression of cyclin D1 [54], and altering the formation of bone structures by increasing the expression of MSX1, MSX2, and DLX5 [55]. Furthermore, ZA can influence the expression of VEGF and ANG-1 in OB-like cells [56].

On the contrary, AL effects were more positive on OB-like cells. The data demonstrated that AL promoted the differentiation of mesenchymal cells into osteoblasts [57–59], as well as increased the proliferation and maturation of osteoblasts [60, 61]. Additionally, the upregulation of BMP2, OCN, and COL1 [59, 61] was observed, as well as that of OSX, RUNX2, OPG, and COL1A1 markers [62]. Negative effects were also presented in a few studies, which showed that AL can decrease the expression of RUNX2, ALP, COL1A1, OSC, BMP2, BMP7, and VEGF [52, 56]. Additionally, it was found to reduce cell proliferation, increase the percentage of cells in the G0/G1 phase, and decrease those in the G2/M phase [63]. Furthermore, AL can inhibit IL-6 [64].

The studies that explored the effects of PAM [27, 39, 40, 63, 65–67] presented ambiguous data. Some studies have shown that higher concentrations of PAM affect cell proliferation [63], decrease cell viability, downregulate RANKL, OPN, and COL1A1 expression, and increase the number of apoptotic cells [27, 40, 66]. On the other hand, other studies [39, 65, 67] demonstrate that PAM can promote the proliferation and maturation of osteoblasts, including increasing the expression of markers such as RUNX2 and OSX.

Some papers have demonstrated that CL can increase cell viability; however, its capacity for differentiation is affected [63]. It was also shown that osteogenic markers, including RUNX2, ALP, COL1A1, OSX, OSC, BMP2, BMP7, and VEGF, were downregulated by CL [52]. The use of higher concentrations resulted in decreased cell viability and adhesion [68], which was associated with an increase in apoptosis [27, 39, 63, 66]. Also, CL can inhibit IL-6 production [64]. Only one study reported that CL did not impair viability, proliferation, mineralization, or collagen expression [40]. Despite this, CL can increase cyclin D expression [54] but does not affect MSX1 and MSX2 [55].

Only 4 studies evaluated the effects of IB on OB-like cells. Data demonstrate that IB can reduce viability [27], adhesion, migration [42], and proliferation of OB-like cells by increasing the percentage of cells in the G0/G1 phase and decreasing the G2/M phase [63]. They demonstrated that IB can decrease cyclin D1 expression over time but increase COL1A1 expression [54], as well as increase the expression of MSX1, MSX2, and DLX5 over time [55].

The effect of RS was only evaluated by Im et al. (2004) [60], and data demonstrate enhanced viability, proliferation, and mineralization regardless of the concentration used. Similarly, OLP was investigated by Mathov et al. (2001) [65], which demonstrated increased calcium influx and ERK1/2 phosphorylation. Finally, 99Tc-MDP enhanced osteoblast proliferation, differentiation, and matrix mineralization. Additionally, OPG and BMP expression were increased, while RANKL expression was decreased [69].

### Bias analysis

**Fig. 2:**
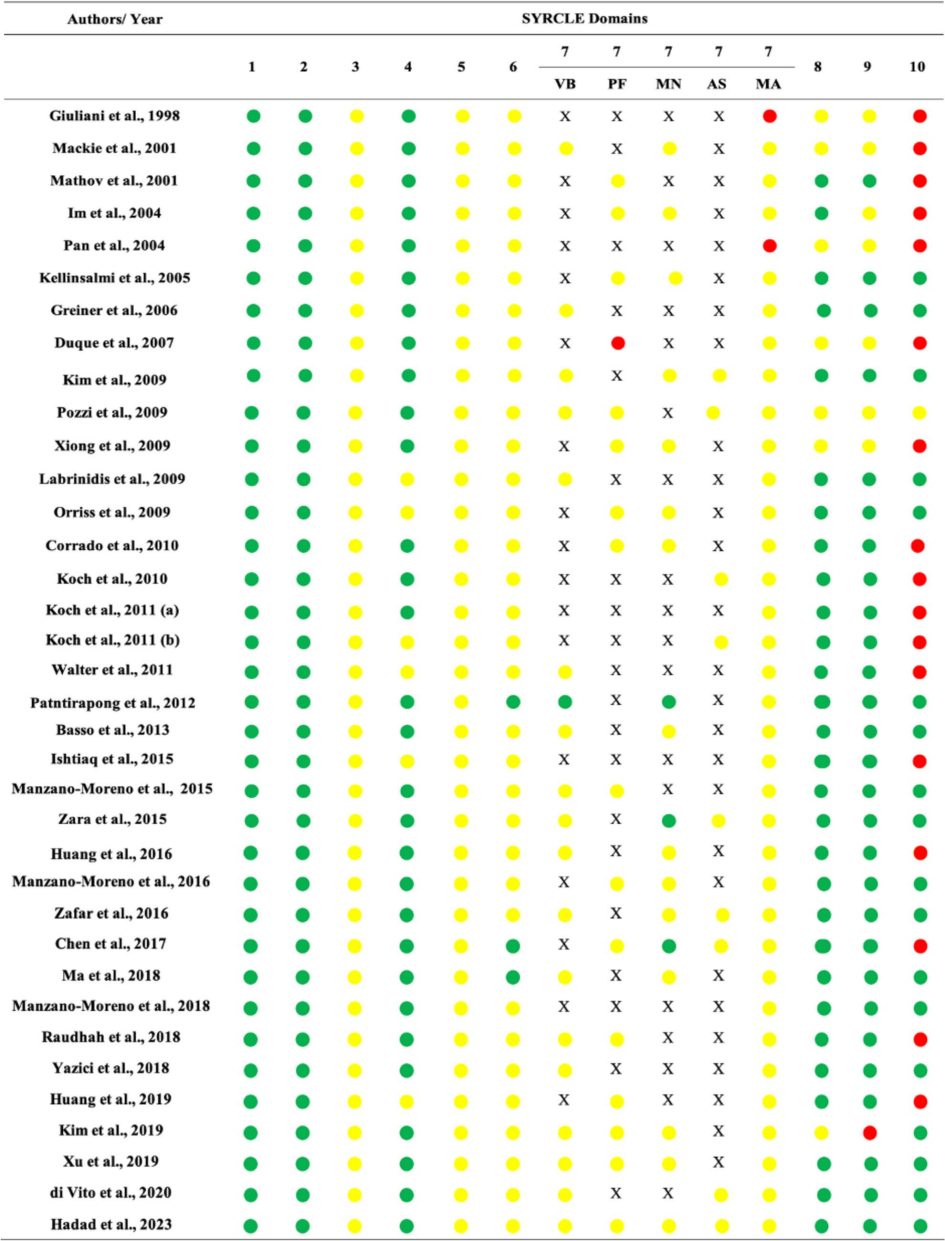
Risk of bias collected by modifying SYRCLE risk of bias tool. 1. Was the allocation sequence adequately generated and applied?; 2. Were the groups similar at baseline, or were they adjusted for confounders in the analysis?; 3. Was the allocation to the different groups adequately concealed?; 4. Were the animals randomly housed during the experiment? (Modified version used: *Did the researcher perform randomization during the experiment?*); 5. Were the caregivers and/or investigators blinded from knowledge of which intervention each animal (groups) received during the experiment? (Modified version used: *Were the investigators blinded from knowledge of which intervention each well/cell received during the experiment?*); 6. Were animals selected at random for outcome assessment? (Modified version used: *Were cells/well/ or plates selected at random for outcome assessment?*); 7. Was the outcome assessor-blinded?; 8. Were incomplete outcome data adequately addressed?; 9. Are reports of the study free of selective outcome reporting?; 10. Was the study apparently free of other problems that could result in high risk of bias?. *VB* viability assay, *PF* proliferation assay, *MN* mineralization assay or ALP activity assay, *AS* adhesion and spreading assay, *MA* markers and other analysis, *red circle* high risk of bias, *yellow circle* unclear information, *green circle* low risk of bias, *X* outcome not evaluated, *SYRCLE* Systematic Review Center for Laboratory animal Experimentation

All the biased information regarding bias was organized and shown in Fig. 2. The studies generally failed to clearly describe allocation and randomization during the experiments. Additionally, blinding was not frequently observed in this review. Some studies presented a high risk of bias due to the absence of statistical analysis of the data [54], failure to compare the experimental groups with the control [55, 60], proposing certain analyses but only partially presenting the data [26, 49, 57, 61, 64, 66, 68], and altering the initially proposed methodology without any justification [27, 33, 56, 65, 67, 69].

## Discussion

The main mechanism of action of BPs (whether N-BPs or NN-BPs) is in osteoclastic cells [22], achieved by incorporating non-hydrolyzable analogs of adenosine triphosphate (ATP) [70] or inhibiting FPP synthase and blocking the prenylation of small GTPases in the mevalonate pathway of cholesterol synthesis [71]. However, this review demonstrated that this drug also acts on other cells and tissues beyond its effects on osteoclasts. Nevertheless, it is important to emphasize that the benefits of therapy with BPs outweigh the risks. Many studies clearly demonstrate these benefits, particularly to control and reduce skeletal-related events (SREs) in patients with bone metastases, breast cancer, and multiple myeloma [72–74].

Although the most common side effects of oral bisphosphonate (BP) use are hypocalcemia and gastrointestinal events, medication-related osteonecrosis of the jaw (MRONJ) may be the most significant side effect associated with its use. According to the American Association of Oral and Maxillofacial Surgery, MRONJ is defined as the presence of exposed bone in the maxillofacial region for a period longer than 8 weeks in patients with no history of radiation therapy or metastatic disease in the jaws who have been treated with antiresorptive agents [14]. Although it has a low incidence (0.01–0.03% in patients treated for osteoporosis and 2–5% in patients undergoing cancer treatment) [75, 76], in dentistry, there is a constant concern about understanding the mechanisms involved in MRONJ, as well as identifying which cells and tissues are affected, given that this condition directly impacts the patient’s quality of life [77].

This concern is the main motivation for conducting this review. However, it can be challenging to draw specific conclusions from the data due to its high variability and ambiguity in effects, particularly in relation to the type of drug used, dose, cell type, and experimental design. Recent studies have begun to elucidate the effects of BPs on osteoblast cells [23] as they can either upregulate or downregulate osteogenesis depending on the concentration used (to clarify, this paper considered all concentrations in molarity [M]), ranging from 10^–9^ to 10^–5^ M [26, 39, 46, 49, 58]. BPs can also modulate the regulation of osteoclastogenic factors from osteoblasts (OB), inhibiting interleukin 6 (IL-6) [64], decreasing RANKL, and increasing OPG and TNF-α converting enzyme (TACE) protein expression in OB-like cells [49, 66].

Moreover, a recently published paper exploring the outcomes of using rodents as an animal model for studying tooth extraction-related medication-related osteonecrosis of the jaw supported the idea that BPs can lead to ONJ-like lesions [78]. However, it was found that many studies used supraphysiologic doses of BPs. A similar observation can be made here, where several studies of BPs concentrations cannot be directly translated to the clinical scenario. This review includes studies that used ZA across a wide concentration range, from as high as 10^–3^ to as low as 10^–12^ M (Table 2). According to the literature, patients undergoing ZA treatment with an intravenous infusion of 4 mg ZA reach a peak plasma concentration, also known as *Cmax*, within 15 min, ranging between 300 and 400 ng/mL (which corresponds to 1.1 µM), with a half-life of more than 192 h [79]. Interestingly, patients undergoing ZA treatment and diagnosed with MRONJ present ZA concentrations ranging from 0.4 to 4.5 µM [80].

The osteoblast lineage originates from mesenchymal stem cells (MSCs) residing in the bone marrow. These cells exhibit multipotency, differentiating into osteoblasts, adipocytes, or chondrocytes based on specific environmental cues [81]. Constituting approximately 4 to 6% of the total bone resident cells, osteoblasts are renowned for their pivotal role in bone formation, deposition, and mineralization [82, 83]. They achieve this primarily by synthesizing and depositing calcium phosphate crystals, such as hydroxyapatite, and extracellular matrix components, including proteoglycans and type 1 collagen [23].

Interestingly, NN-BPs seem not to influence OB proliferation and type 1 collagen (COLA1) expression [54]. The use of AL and RS (doses varying from 10^–12^ M up to 10^–4^ M) stimulated cellular proliferation in HOBs and human osteoblast-like cells (MG-63). Additionally, both drugs induced a high level of ALP and OCN at a concentration of 10^–8^ M [60]. Indeed, similar results were observed by Xiong et al. (2009) [61] using AL in the range of 10^–10^ to 10^–6^ M in MG-63 cells, and they also reported an increase in ALP, BMP2, COL1A1, and OCN. Similar observations were made by Duque et al. (2007) [57] when after 2 weeks of AL exposition (10^–9^ up to 10^–7^ M), human bone mesenchymal stem cells (hBMSCs) expressed a higher level of ALP, a higher amount of mineralization (in a dose-dependent effect), and increased expression of ALN and CBFA1 (core-binding factor subunit alpha-1, which is a critical regulator of osteoblast differentiation).

Doses of AL, ranging from 10^–10^ up to 10^–8^ M, stimulated cellular proliferation and calcium deposition in MG-63 cells after 48 and 72 h in culture [61]. It also upregulated the expression of BMP2, OCN, and COL1A1 after 96 h of culture, with sustained expression after 7 days in culture. Additionally, clodronate (10^–9^, 10^–7^, 10^–5^ M) can significantly stimulate MG-63 osteoblast-like cells proliferation; however, it affects ALP activity (mainly at 10^–9^ M) [84].

AL enhances the viability and proliferation of osteoblasts and bone formation in an osteoporosis female rat model induced by ovariectomy [62]. Additionally, the results demonstrated that AL improves bone loss in osteoporosis through the upregulation of the interferon‑β/signal transducer and activator of transcription 1 pathway (IFN-β/STAT1), which is strongly related to the osteoporosis pathogenesis [85]. Additionally, AL stimulates osteoblast differentiation through the PKA-STAT3 and STAT1 pathways [66]. Additionally, AL enhances the differentiation of bone marrow cells (BMCs) into osteoblasts, significantly increasing ALP and reducing tartrate-resistant acid phosphatase (TRAP) expression [62]. Immunomarkers for osteogenic differentiation, such as osteoclast differentiation factor (ODF), OCN, OSX, RUNX2, OPG, and COL1A1, were higher in the AL group compared to the control [53, 86].

AL (10^–6^ M) promotes osteoblast differentiation and up-regulates ALP in osteoblasts after 48 and 72 h [56]. Additionally, it is noteworthy that AL stimulates the expression levels of mRNAs associated with osteoblast differentiation (OCN, OSX, and RUNX2) and increases the expression of osteoclast differentiation factors (ODF), OPG, and COL1A1. Furthermore, AL regulates bone formation and osteoblast differentiation through the IFN-β/STAT1 pathway.

This same group demonstrated that ibandronate, zoledronate, and clodronate stimulate homeobox protein 1n (MSX1) gene expression in a dose-dependent manner in HOBs [55]. Additionally, the expression of homeobox protein 2 (MSX2) was stimulated by ibandronate and clodronate, primarily by ZA (5 × 10^–5^ M). MSX2 is a component of the BMP signaling pathway, which regulates cell growth, including the formation of new bone cells [87]. However, only ZA and IB enhanced DLX5 expression. DLX5 serves as an early BMP-responsive transcriptional activator, promoting osteoblast differentiation by upregulating various promoters, including ALP, the transcription factor SP7 (essential for bone formation), and the myelocytomatosis oncogene protein (MYC) [88]. It appears that N-BPs enhance bone density as they exhibit higher expression of RUNX2 and OCN in a dose-dependent manner.

Previously published papers have demonstrated that ZA (10^–6^ and 5 × 10^–6^ M) decreases cell viability in MG-63 cells and reduces the expression of OCN and ALP after 14 days [47]. Data suggest that ZA (5 × 10^–5^ M) has a negative effect on HOBs [54], decreasing cyclin D1 expression after 6 days of exposure, which could result in a proliferative impact. High doses (5 × 10^–5^, 5 × 10^–4^ M) of AL, pamidronate, ibandronate, and clodronate can also reduce the proliferation of MG-63 cells by arresting the cell cycle in the G0/G1 phase [63]. This is achieved through an increase in the expression of cyclin-CDK inhibitors p21 and p27 and an increase in the expression of annexin V [45, 63]. Also, the N-BPs, particularly pamidronate and zoledronate, affect cell viability and cell migration of osteoblasts by inducing apoptosis, as demonstrated by TUNEL-Assay [27, 89]. Additionally, after 72 h of pamidronate (4.5 × 10^−5^ M) exposition, HOBs viability can decrease significantly compared to control, but RUNX2 and OSX increased significantly over time [67].

Olpadronate, pamidronate, and etidronate (10^−7^ M) increased cell proliferation, and data suggested that this outcome can be mediated by the activation of extracellular signal-regulated kinase (ERK) and the influx of calcium from the extracellular space [65]. However, ZA (5 × 10^–6^ M) was observed to induce apoptosis and mitochondrial oxidative stress and decrease calcium signaling in SAOS-2 cells [48]. ERKs are members of the mitogen-activated protein kinases family (MAPKs), and their function is related to regulating cell growth, proliferation, and differentiation [90].

ZA decreases ALP expression and calcium deposition, an important phenotypic marker for the early differentiation of osteoblast, even in low dosages (0.01 × 10^–6^ and 10^–6^ M) [33]. Also, they can critically affect the expression of COL1A1, OCN, RUNX2, and BMP2, but not MAPK p38 and ERK1/2, which is essential for skeletal development and homeostasis [63, 68], also observed by Patntirapong et al. (2012) [51] using various concentrations (0, 0.01 × 10^–6^, 0.1 × 10^–6^, 10^–6^, 5 × 10^–6^, 10 × 10^–6^, 50 × 10^–6^, and 100 × 10^–6^ M). It is important to highlight that besides influencing the osteogenic potential, N-BPs (ZA, range varying from 10^–10^ up to 10^–6^ M) and AL (10^–7^ and 10^–6^ M) suppress osteoblastic production of vascular endothelial growth factor (VEGF) and angiopoietin-1 (ANG-1), at higher, non-toxic concentrations [56]. The chronic ZA exposure (in concentrations > 10^–3^ M) demonstrated gradual downregulation of messenger RNA (mRNA) expression level for ALP, COL1, and RUNX2; however, there were no significant alterations for OCN in osteogenic differentiation of periodontal ligament stem cells [32].

Even if it does not completely inhibit differentiation, studies have shown that ZA (10^–6^ M) can delay the osteoblastic differentiation process in HOBs, decreasing IL-6 and OCN [43]. However, Hadad et al. (2023) [25] demonstrated that ZA, particularly at a concentration of 5 × 10^–6^ M, adversely affects the differentiation of hBMSCs toward the osteoblastic lineage by reducing cell proliferation, impairing adhesion, spreading, and downregulating RUNX2 expression. RUNX2 is essential for the differentiation of osteoblast progenitor cells into pre-osteoblasts; its expression is positively regulated in pre-osteoblasts, and they exhibit ALP activity [82, 83]. Additionally, the subsequent differentiation of pre-osteoblasts into mature osteoblasts is characterized by an increase in OSX expression and the secretion of bone matrix proteins, including OCN, BSP I/II, and type I collagen [83, 91].

This ultimately results in reduced ALP activity and downregulated expression of ALPL and COL1A1 genes, which are markers of later phases of osteogenesis [42]. ZA (5 × 10^–5^ M) can modify osteoblast morphology, in addition to affecting tenascin C (TNC), which impairs adhesion. TNC is fundamental in osteoblast migration and adhesion, and its lower expression can be related to reduced osteoblast differentiation and bone formation, as mineralization [92].

It was also found that ZA (10^–7^ and 5 × 10^–7^ M) decreases nodule formation in HOBs after 72 h of exposure and significantly increases the expression of caspase-3/7, leading to a 50% reduction in migration capacity and viability [44]. It was also demonstrated by Yazıcı et al. (2018) [48] that ZA (5 × 10^–6^ M) decreases calcium signaling and increases apoptosis (caspases-3 and 9) and by Labrinidis et al. (2009) [41] showing that ZA (10^–5^ and 10^–4^ M) leads to an inhibition of K-HOS cells, in a dose-dependent manner, resulting in marked cytotoxicity and an increase of caspase-3. In the meantime, it was demonstrated that ZA (10^−4^ M) can inhibit dickkopf-1 (DKK1) in breast cancer cells, which affects the Wnt/β-catenin signaling axis [93]. DDK1 plays a central role in bone homeostasis by inhibiting beta-catenin-dependent Wnt signaling [94].

Osteoblasts are also involved in regulating osteoclasts, including their differentiation and activity. Since osteoblast lineage cells express a membrane-resident protein known as RANKL, which binds with the RANK expressed on osteoclast precursors and is involved in the differentiation of osteoclast precursors into osteoclasts. RANKL also binds to RANK of mature osteoclasts, stimulating bone resorption [23, 95, 96]. Osteoblasts also express the secreted OPG, which acts as a receptor for RANKL and inhibits osteoclast differentiation [23, 95, 97–99]. The control of osteoclastogenesis by osteoblasts underscores the importance of these cells in modulating bone resorption. In addition, osteoblasts express numerous other molecules involved in regulating osteoclastogenesis [23], such as tumor necrosis factor-alpha (TNF-α), interleukin-1 (IL-1), and macrophage colony-stimulating factor (M-CSF) [100–102].

It is notable that an in vitro study of HOBs and MG-63 cell lines suggests that low BP doses can significantly impact the expression of genes essential for osteoblast growth and differentiation, as well as genes involved in regulating osteoblast–osteoclast interaction, possibly by increasing transforming growth factor beta1 (TGF-β1) production [52]. Similarly, findings [50] demonstrated that small doses of ZA can reduce migration (5 × 10–5 M), a significant decrease in the procollagen I (3 × 10^–5^ M), OPG expression (10^–5^ M), and RANKL (10^–5^ M). In addition, new bisphosphonates, such as technetium 99 m-methyl diphosphonate (99Tc-MDP, 10^–8^ M), have been shown to increase osteoblast proliferation and differentiation, as evidenced by the overexpression of BMP2 and ALP [69]. Notably, 99Tc-MDP induced osteoblasts to express OPG, resulting in an increased ratio of OPG/RANKL, which can inhibit osteoclast differentiation and favor bone formation [103].

## Conclusion

This review provides a comprehensive overview of the influence of BPs on osteoblasts, emphasizing their molecular mechanisms, therapeutic implications, and potential adverse effects in the treatment of metabolic bone disorders. The effects of BPs on osteoblast cell lineages appear to vary depending on the type of drug used, as well as the concentration and duration of use, which may lead to either stimulation or inhibition of osteogenesis.

The impact of BPs on osteoblasts remains a topic of controversy regarding bone healing. Bisphosphonates have a predominantly positive effect in preventing skeletal-related events (SREs) and presenting several clinical benefits, as discussed in this review. Studies have shown that nitrogen-containing BPs impair osteoblast cells and their differentiation into osteoblast lineages during bone healing. Considering the differences in the effects of N-BPs versus non-N-BPs on osteoblasts, cellular responses varied in terms of proliferation, gene expression, and mineralization capacity.

## Data Availability

Not applicable.
